# A Fulminant Case of Incontinentia Pigmenti Presenting With Early Neuro-Ophthalmologic Manifestations

**DOI:** 10.7759/cureus.100104

**Published:** 2025-12-26

**Authors:** Manon Gillet, Morgane Jankowski, Jean Radermacher, André Mulder

**Affiliations:** 1 Pediatrics, Catholic University of Louvain, Brussels, BEL; 2 Pediatric Neurology, Catholic University of Louvain, Brussels, BEL; 3 Anatomic Pathology and Cytology, Christian Hospital Center (CHC) Montlégia Clinic, Liège, BEL; 4 Pediatrics, Christian Hospital Center (CHC) Montlégia Clinic, Liège, BEL

**Keywords:** early diagnosis, ikbkg gene, incontinentia pigmenti, neuro-ophthalmologic manifestations, status epilepticus

## Abstract

Incontinentia pigmenti (IP) is a rare, X-linked dominant, multisystemic genetic disorder. We report the case of a female neonate presenting with vesiculobullous and erythematous cutaneous lesions involving the hands and feet. Concurrently, she exhibited repetitive flexion movements of all four limbs, which rapidly progressed to status epilepticus. The clinical course was rapidly unfavorable, marked by diffuse retinal ischemia and cerebral palsy.

This patient's clinical history illustrates the potential severity of the neurological and ophthalmologic manifestations of IP. It underscores the need to consider IP in the differential diagnosis of cutaneous lesions associated with neurological symptoms in female infants. Early recognition is essential to ensure close monitoring and initiate appropriate multidisciplinary management.

## Introduction

Incontinentia pigmenti (IP) is a rare, X-linked dominant, multisystem genetic disorder. It is caused by a pathogenic variant in the IKBKG gene, which encodes a protein essential for the activation of NF-κB. This transcription factor plays a central role in inflammation, immunity, and cell survival [1-5]. The main systems affected include the central nervous system, eyes, skin, teeth, and hair. Cutaneous involvement often represents an early and key diagnostic feature.

Neurological complications may range from mild developmental delay to refractory epilepsy, with early intervention significantly improving the child’s prognosis. Ophthalmologic manifestations can vary from mild strabismus to retinal detachment, the latter representing the most severe outcome, which can often be prevented through timely detection and management. Together, these neurological and ophthalmologic features largely determine disease severity and highlight the critical importance of early diagnosis and multidisciplinary care [1-5]. Diagnosis is based on clinical criteria, with genetic testing reserved for confirmation or for atypical presentations.

## Case presentation

**Figure 1:**
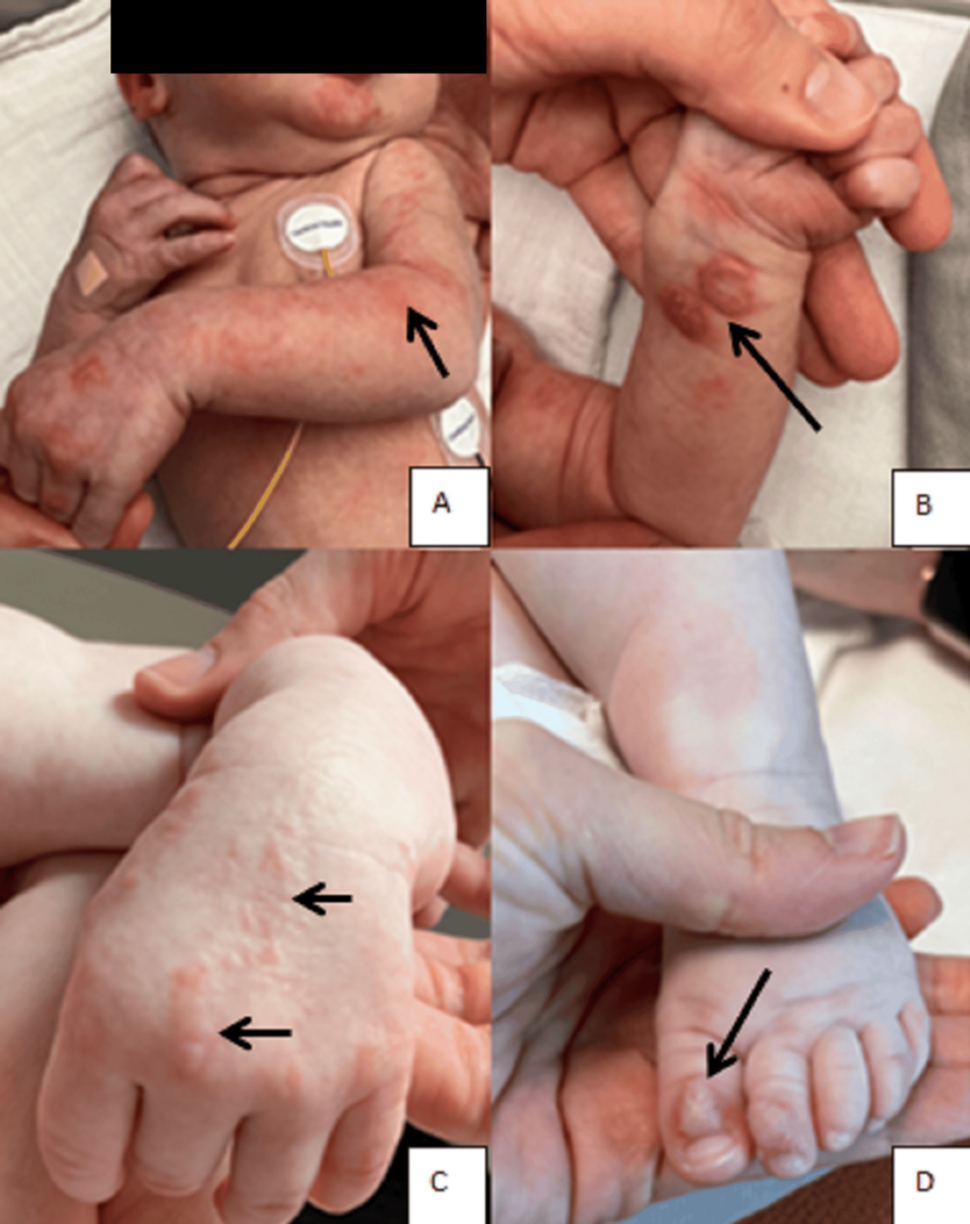
Skin lesions in a newborn with IP. Skin lesions at the erythematous stage (A) and vesiculobullous stage (B). Evolution toward a stage of hyperpigmentation following Blaschko’s lines (C), then progression to a verrucous stage (D). IP: Incontinentia pigmenti

A six-day-old female neonate was admitted into the Intensive Care Unit for status epilepticus, associated with vesiculobullous and erythematous lesions on the hands and feet (Figures 1A-1B).

She was born at term, with no perinatal complications or identifiable maternal-fetal infectious risk factors. Her initial neonatal course was unremarkable. Family history revealed no parental consanguinity. There were no notable familial medical conditions.

Given the clinical presentation, an infectious etiology was initially suspected. Empirical antibiotic therapy with cefotaxime and amoxicillin, along with acyclovir, was initiated. Status epilepticus was controlled with a triple antiepileptic regimen consisting of phenytoin, phenobarbital, and a loading dose of levetiracetam.

Extensive bacteriological and virological investigations (including cerebrospinal fluid, urine, blood, and nasopharyngeal samples) were negative. Following the exclusion of infectious causes, additional autoimmune, coagulation, and metabolic evaluations were also performed and found to be within normal limits (Tables 1-2).

**Table 1:** Quantitative laboratory data for the patient. CMV: Cytomegalovirus; IgG: Immunoglobulin G; IgM: Immunoglobulin M; HSV-1: Herpes simplex virus type 1; HSV-2: Herpes simplex virus type 2; APTT: Activated partial thromboplastin time; INR: International normalized ratio; FVII: Coagulation factor VII; FVIII: Coagulation factor VIII; FIX: Coagulation factor IX; IgA: Immunoglobulin A; CD3: Cluster of differentiation 3; CD4: Cluster of differentiation 4; CD8: Cluster of differentiation 8; CD19: Cluster of differentiation 19; NK: Natural killer

Test	Values of the patient	Reference values
Bacterial serology		
CMV IgG	70U/mL	IgG positive if >12U/mL
CMV IgM	3U/mL	IgM positive if >12U/mL
HSV1 IgG	>30000U/mL	IgG positive if >12U/mL
HSV1 IgM	0U/mL	IgM positive if >12U/mL
HSV 2 IgG	>30000U/mL	IgG positive if >12U/mL
HSV2 IgM	0U/mL	IgM positive if >12U/mL
Parvovirus B19 IgG	27U/mL	IgG positive if >15U/mL
Parvovirus B19 IgM	3U/mL	IgM positive if >15U/mL
Toxoplasmose IgG	79U/mL	IgG positive if >15U/mL
Toxoplasmose IgM	0.2U/mL	IgM positive if >1.5U/mL
Coagulation		
APTT	35.3s	35-55s
INR	1.1	1-1.3
Fibrinogen	2.09g/L	1.5-3.5g/L
Prothrombin time	12.9s	12-18s
FVII, FVIII, IX	110%, 120%, 53%	20-60%, 50-150%, 30-60%
Protéine C,S	35-38%	20-40%, 30-50%
Markers of immunity		
IgG	7.49g/L	4-12g/L
IgA	<0.15g/L	<0.05g/L
IgM	0.14g/L	0.2-0.7g/L
Ratio CD4/CD8	2.7	1.5-3.5
CD3+	80%	55-75%
CD4+	57%	35-55%
CD8+	21%	15-25%
B-lymphocyte - CD19	16%	15-35%
NK-CD16	4%	5-15%
Lumbar puncture		
Protein	2.026g/L	1-1.5g/L
Blood glucose	53mg/dL	50-80mg/dL
Blood cell	100/mm^3^	<5/mm^3^
White cell	7/mm^3^	0-20/mm^3^
Urine		
Blood cell	10/mcgL	<5-10/mcgL
White cell	4/mcgL	<0-5/mcgL

**Table 2:** Qualitative laboratory results of the patient. PCR: Polymerase chain reaction; HSV: Herpes simplex virus; HZV: Human herpesvirus 3

Test	Values of the patient	Reference values
Nasopharyngeal aspirate		
PCR Adenovirus	Negative	Negative if undetectable
PCR Parainfluenza	Negative	Negative if undetectable
PCR Metapneumovirus	Negative	Negative if undetectable
PCR Enterovirus	Negative	Negative if undetectable
Lumbar puncture		
PCR *Candida*	Negative	Negative if undetectable
PCR HSV 1 and 2	Negative	Negative if undetectable
PCR enterovirus	Negative	Negative if undetectable
PCR HZV	Negative	Negative if undetectable
PCR multiplex for Escherichia coli	Negative	Negative if undetectable
PCR multiplex for *Listeria*	Negative	Negative if undetectable
PCR multiplex for *Haemophilus influenzae*	Negative	Negative if undetectable
PCR multiplex for *Streptococcus agalactiae*	Negative	Negative if undetectable
PCR multiplex for *Neisseria meningitidis*	Negative	Negative if undetectable
PCR multiplex for *Streptococcus pneumoniae*	Negative	Negative if undetectable
Bacteria culture	Negative	Negative if undetectable
Urine		
Bacteria culture	Negative	Negative if undetectable
Metabolic investigation		
Amino acid profile in blood	Within normal limits	No abnormalities detected
Amino acid profile in urine	Within normal limits	No abnormalities detected

**Figure 2:**
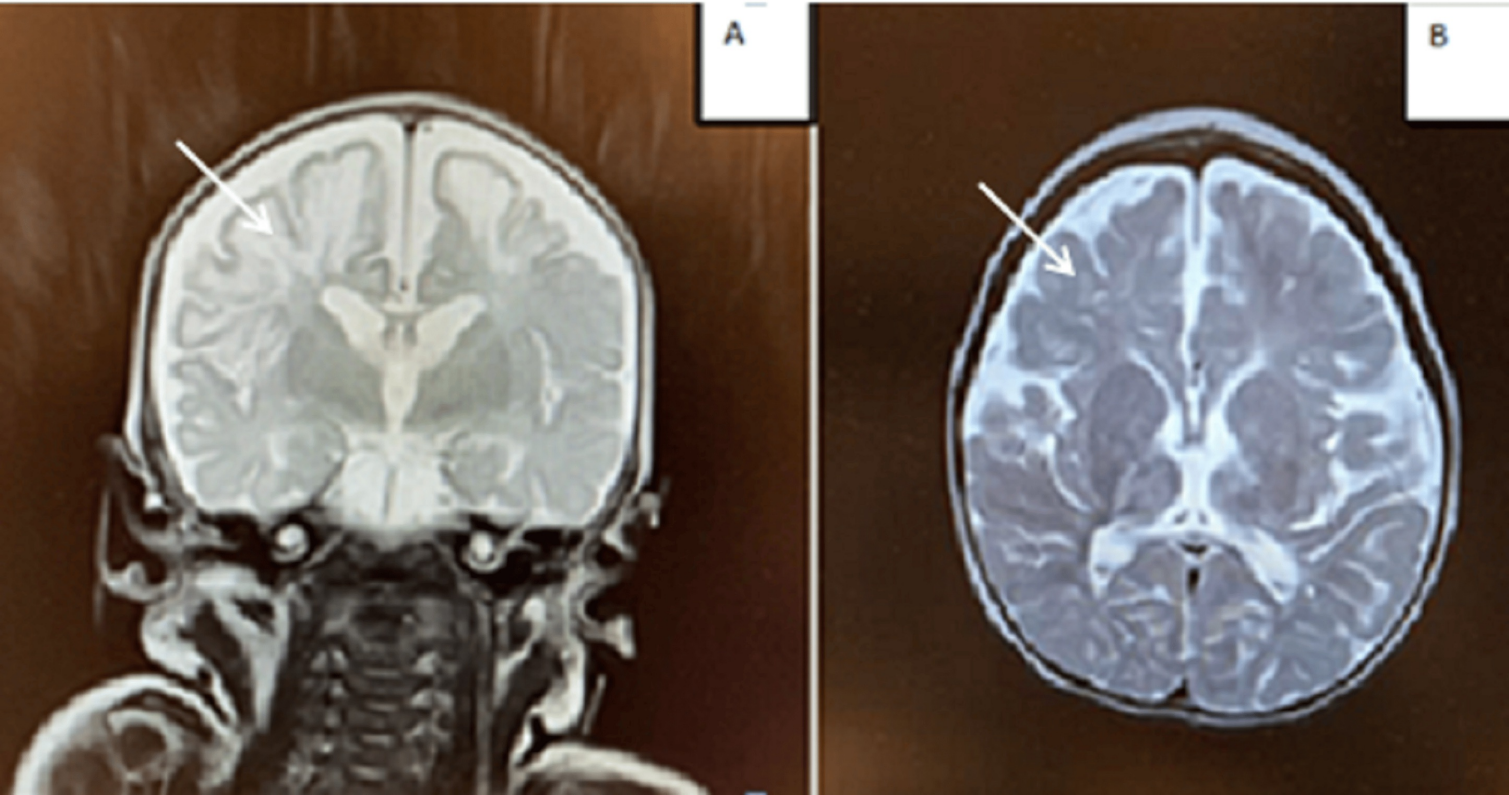
Brain MRI in T2 sequence. (A) Coronal section at one week of age showing diffuse hyperintensity of the white matter (arrow). (B) Axial section at 25 months of age showing persistent diffuse hyperintensity of the white matter with very limited myelination (arrow).

Given the persistently negative biological and urinary work-up and the ongoing seizures, a brain MRI was performed. The MRI showed bilateral supra- and infratentorial ischemic and hemorrhagic lesions, affecting both cortex and subcortical white matter, as well as involvement of the corpus callosum and basal ganglia (Figure 2A). A fundoscopic examination performed in this context initially revealed normal findings.

Considering the cerebral lesions in association with the cutaneous findings, a genetic analysis targeting IP was also performed. Whole-exome sequencing did not reveal any pathogenic variants.

Since no definitive etiology was identified and the clinical evolution became favorable, the patient was discharged after three weeks of hospitalization under phenytoin and phenobarbital treatment.

**Figure 3:**
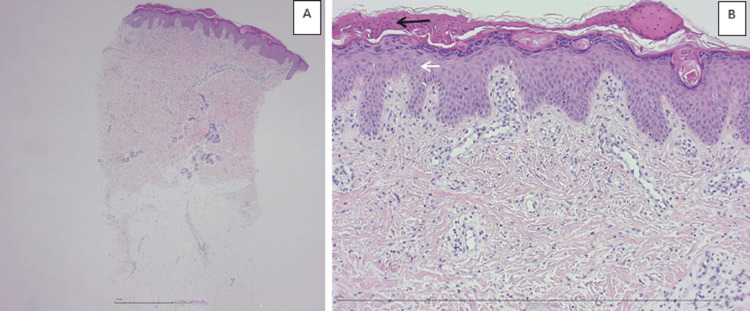
Microscopic analysis of a skin biopsy in a newborn with IP at low (A) and high magnification (B). This skin biopsy shows hyperkeratotic parakeratosis (black arrow) and eosinophilic spongiosis (white arrow). IP: Incontinentia pigmenti

At the age of two months, following routine vaccinations, the patient presented with a new inflammatory skin flare-up, characterized by hyperpigmented lesions and pseudo-verrucous scarring along Blaschko’s lines (Figures 1C-1D). Clinical examination revealed significant axial hypotonia and poor visual tracking. Blood analysis showed eosinophilia and moderate T-cell lymphopenia. A skin biopsy revealed eosinophilic spongiosis and parakeratotic hyperkeratosis, findings typical of IP (Figures 3A-3B).

Following this recurrence of skin manifestations, a specific long-range PCR targeting the IKBKG gene was performed. It revealed a pathogen heterozygous deletion of exons 4 to 10 of the gene. Maternal testing confirmed the absence of this deletion, suggesting a likely de novo event in the patient. This deletion affects a large segment of the gene, which shares more than 99% homology with a nearby pseudogene, making it undetectable by standard next-generation sequencing (NGS) techniques.

At the age of five months, while still on phenobarbital, the patient developed infantile spasms, requiring a new triple therapy regimen consisting of vigabatrin, levetiracetam, and tetracosactide. She subsequently progressed to a clinical picture consistent with cerebral palsy, presenting as spastic tetraparesis. At the age of 25 months, a follow-up MRI was performed and revealed a diffuse hyperintensity of the white matter with very limited myelination (Figure 2B).

On the ophthalmologic side, despite close monitoring and an initially normal fundus examination at the age of two months, the patient developed advanced and diffuse retinal ischemia in the left eye. This required panretinal photocoagulation sessions, intravitreal ranibizumab injections, and treatment with anti-vascular endothelial growth factor (anti-VEGF) agents.

In summary, the patient initially presented with cutaneous lesions and seizures associated with cerebral lesions; extensive infectious, autoimmune, metabolic, and genetic evaluations were negative, and only after the emergence of a second wave of cutaneous lesions was a skin biopsy performed, providing additional support for the suspicion of IP and prompting a repeat genetic analysis.

## Discussion

IP is a rare, multisystemic ectodermal dysplasia with X-linked dominant inheritance [1-5]. IP is caused by a pathogenic variant in the IKBKG gene, located on chromosome Xq28. This gene encodes a subunit of the IKK protein complex, which regulates the activity of the nuclear transcription factor NF-κB. NF-κB plays a critical role in immune responses, inflammation, and cell survival. It protects cells against apoptosis, particularly that induced by tumor necrosis factor-alpha (TNF-α), and contributes to the integrity of cerebral and retinal endothelial cells, as well as the blood-brain barrier. In IP, mutated cells are therefore particularly prone to apoptosis [1,2].

In our patient, as roughly speaking 80% of cases, a deletion involving exons 4 to 10 of IKBKG was identified [1-5]. Whole exome sequencing (WES) using NGS may be required to support the diagnosis. However, when NGS is inconclusive - as is the case for our patient - a specific PCR targeting IKBKG should be performed. The presence of a non-functional pseudogene, IKBKGP1, which shares high homology with IKBKG, complicates mutation analysis using conventional methods [4].

Once IP is suspected, genetic counseling should be offered to the family. In our case, the deletion was not identified in either parent, suggesting a de novo mutation, which occurs in approximately 75% of cases [5]. The estimated prevalence of IP is 1 in 40,000 to 50,000 live births [5], corresponding to an average of 27 new cases reported worldwide annually [2]. The condition occurs almost exclusively in females [1-5], as it is generally lethal in males. However, 28 male cases have been reported in the literature, mostly associated with Klinefelter syndrome or post-zygotic mosaicism [1-5].

IP exhibits significant phenotypic variability, largely due to the process of skewed X-chromosome inactivation in females, which results in a variable distribution of functionally different cell populations [4]. Clinical manifestations may involve the skin, eyes, central nervous system, hair, teeth, and nails [1-5]. The diagnosis of IP is primarily clinical and is based on criteria first proposed by Landy and Donnai in 1993 [1,3,5]. Major diagnostic criteria include typical skin changes, dental agenesis or delayed tooth eruption, and a characteristic genetic rearrangement (deletion of exons 4-10) [4]. Minor criteria include eosinophilia, hair and nail abnormalities, ocular involvement, and the typical histological features observed in skin lesions.

In the presence of a first-degree affected relative, only one major or two minor criteria are needed for diagnosis. In the absence of a family history - as is the case for our patient's family background - the combination of one major and one minor criterion is required. Ocular involvement is not part of the current diagnostic criteria, despite its potential severity. In our case, the retinal disease was aggressive and highlights the importance of early ophthalmologic screening and intervention to prevent long-term complications. The exclusion of ocular and neurological findings from the major diagnostic criteria may be due to their clinical heterogeneity, making them more difficult to standardize and incorporate into diagnostic algorithms.

Cutaneous involvement

Cutaneous involvement is pathognomonic of IP. It typically progresses through four distinct stages, although these may appear simultaneously, as observed in our patient. The first stage usually manifests within the first week of life and typically resolves within four to six months. It is characterized by vesiculobullous and erythematous lesions [4], observed in nearly 100% of patients, and predominantly located on acral areas [2]. This stage may be mistaken for a bacterial skin infection, and empiric treatment should be initiated [1]. Once an infectious workup is negative, IP should be considered among the differential diagnoses [1,4]. Skin biopsy typically reveals eosinophilic spongiosis and dyskeratotic keratinocytes, as seen in our patient [1].

As illustrated in our case, cutaneous flares may recur during viral infections with fever spikes or following vaccinations [1]. In contrast to most reports in the literature, we observed that topical corticosteroids and antiseptic lotions were effective in stabilizing the skin lesions during these flare-ups [1-4]. No data are currently available regarding the need for analgesia during cutaneous flares in IP. However, our patient experienced multiple episodes of painful exacerbations, which required administration of level I and II analgesics to relieve significant discomfort. The second stage typically occurs within the first two months of life and is characterized by verrucous lesions following the lines of Blaschko. Histologically, these lesions show epidermal hyperplasia on biopsy. The third stage typically predominates during the first two years of life. It is characterized by linear hyperpigmentation, as observed in our patient at the age of 14 months [1-5]. The fourth stage is characterized by residual hypopigmentation, which often persists throughout life [1-5].

The pathophysiology underlying these skin lesions remains a topic for debate. However, apoptosis of mutated cells, their replacement by healthy cells, and the associated secretion of inflammatory cytokines are believed to be key mechanisms involved in the evolution of the cutaneous manifestations [1].

Neurological involvement

Neurological manifestations of IP are highly heterogeneous and include epilepsy, psychomotor delay, intellectual disability, microcephaly, and learning difficulties. Approximately one-third of patients with IP develop neurological lesions [1,4]. Severe neurological involvement is more frequently observed during the first weeks of life, as in our patient, who presented with acute encephalopathy and ischemic-hemorrhagic lesions a few days after birth. MRI findings typically include periventricular white matter abnormalities characterized by T2-weighted hyperintensities, as well as more pronounced cortical lesions, with areas of acute diffusion restriction lacking a vascular distribution, contrast enhancement, microhemorrhages, and/or focal cortical atrophy [1,4]. Early brain MRI is recommended when IP is suspected, particularly in the presence of seizures or acute neurological symptoms. The differential diagnosis includes hypoxic-ischemic encephalopathy, stroke, and infectious encephalitis [6].

Some centers have reported favorable outcomes with systemic corticosteroids in cases of cerebral edema, although data remain limited. Despite these limitations, this approach represents a promising avenue for future research in larger cohorts. With regard to epilepsy management, there are currently no specific recommendations for the use of antiepileptic drugs in patients with IP [1,3,4].

Ophthalmological involvement

Ophthalmologic manifestations include both retinal and non-retinal abnormalities, such as microphthalmia, strabismus, cataract, vitreous hemorrhage, optic nerve abnormalities, and nystagmus [1-5]. The majority of individuals with IP have normal vision; however, ocular manifestations often result from vascular abnormalities that typically worsen the prognosis and can lead to blindness [2]. The retinal involvement observed in our patient was secondary to mechanisms of retinal ischemia and neovascularization, ultimately causing retinal detachment. Our patient had a normal ophthalmological examination at the age of two months, but by four months, severe and extensive retinal ischemia had developed.

Early intervention with panretinal photocoagulation and administration of VEGF analogs should be initiated promptly to prevent progression of retinal lesions [1,4].

Dental, nail, and hair involvement

Dental abnormalities commonly include agenesis, conical-shaped teeth, and delayed eruption with persistence of deciduous teeth [1-4]. Nail involvement mainly consists of nail dystrophy and onychogryphosis, with less frequent occurrences of papillomas or subungual cysts [1-4]. Hair involvement in IP is less common than dental or nail abnormalities but can affect hair texture, density, and quality, potentially leading to alopecia [1-4]. Given the potential severity of complications, early screening is essential. A comprehensive follow-up program addressing neurological, cutaneous, dental, and ophthalmologic aspects is clearly recommended for patients with IP, as emphasized by the multidisciplinary recommendations of the European network [4].

Treatment is symptomatic and tailored to the type of involvement. The NF-κB pathway, whose dysregulation plays a central role in IP, represents a promising therapeutic target. Pharmacological strategies aimed at modulating the NF-κB pathway are currently being investigated in various pathological contexts [7] and could potentially be adapted for the treatment of IP. Preclinical studies in murine models seek to demonstrate that the introduction of a functional copy of the IKBKG gene may attenuate certain clinical manifestations, particularly neurological symptoms [8].

Although these strategies remain experimental, they offer innovative perspectives for the management and potential cure of IP. This case highlights the need to consider IP in female neonates presenting with concomitant cutaneous and neurological signs. Early diagnosis, even in the presence of an initially negative genetic test, and prompt multidisciplinary management may improve neurological and ophthalmologic outcomes. Clinicians should remain vigilant to ensure timely diagnosis and appropriate care.

## Conclusions

IP is a multisystem disorder with potentially severe and irreversible complications. It should be considered in female neonates presenting with erythematous vesiculobullous skin eruptions and neurological symptoms. Cutaneous involvement remains the cornerstone for diagnosis, while ophthalmologic and neurological manifestations largely determine disease severity. The overall pathophysiology is not yet fully understood, though inflammation, neovascularization, and increased susceptibility to apoptosis appear to play central roles across affected systems. In our patient, these included status epilepticus, retinal ischemia, and subsequent cerebral palsy, illustrating the potential severity of IP. Treatment is symptomatic and tailored to organ involvement. Systemic corticosteroids have been proposed for neurological manifestations, though efficacy is limited, and experimental strategies targeting the NF-κB pathway, including the introduction of a functional IKBKG gene, may offer future therapeutic options.
